# SeMPI 2.0—A Web Server for PKS and NRPS Predictions Combined with Metabolite Screening in Natural Product Databases

**DOI:** 10.3390/metabo11010013

**Published:** 2020-12-29

**Authors:** Paul F. Zierep, Adriana T. Ceci, Ilia Dobrusin, Sinclair C. Rockwell-Kollmann, Stefan Günther

**Affiliations:** 1 Institute of Pharmaceutical Sciences, Albert-Ludwigs-Universität Freiburg, Hermann-Herder-Straße 9, 79104 Freiburg, Germany; paul.zierep@pharmazie.uni-freiburg.de (P.F.Z.); idobrusin@posteo.de (I.D.); rokoko.sinc@outlook.com (S.C.R.-K.); 2 Department of Cellular, Computational and Integrative Biology (CIBIO), University of Trento, Via Sommarive 9, Povo, 38123 Trento, Italy; adriana.ceci92@gmail.com

**Keywords:** secondary metabolites, natural compounds, machine learning, nonribosomal peptides, polyketides

## Abstract

Microorganisms produce secondary metabolites with a remarkable range of bioactive properties. The constantly increasing amount of published genomic data provides the opportunity for efficient identification of biosynthetic gene clusters by genome mining. On the other hand, for many natural products with resolved structures, the encoding biosynthetic gene clusters have not been identified yet. Of those secondary metabolites, the scaffolds of nonribosomal peptides and polyketides (type I modular) can be predicted due to their building block-like assembly. SeMPI v2 provides a comprehensive prediction pipeline, which includes the screening of the scaffold in publicly available natural compound databases. The screening algorithm was designed to detect homologous structures even for partial, incomplete clusters. The pipeline allows linking of gene clusters to known natural products and therefore also provides a metric to estimate the novelty of the cluster if a matching scaffold cannot be found. Whereas currently available tools attempt to provide comprehensive information about a wide range of gene clusters, SeMPI v2 aims to focus on precise predictions. Therefore, the cluster detection algorithm, including building block generation and domain substrate prediction, was thoroughly refined and benchmarked, to provide high-quality scaffold predictions. In a benchmark based on 559 gene clusters, SeMPI v2 achieved comparable or better results than antiSMASH v5. Additionally, the SeMPI v2 web server provides features that can help to further investigate a submitted gene cluster, such as the incorporation of a genome browser, and the possibility to modify a predicted scaffold in a workbench before the database screening.

## 1. Introduction

Microorganisms, such as bacteria and fungi, have always been subject to evolutionary pressure. The law of “survival of the fittest” has led to a remarkable diversity of strategies to overcome competitors. At the level of biosynthesis, microorganisms “learned” to produce a vast number of natural products that help them to survive [1]. These secondary metabolites (SMs) often possess biological activities, which can be exploited for pharmaceutical purposes. The molecular machinery for the production of SMs is encoded in gene assemblies organized in biosynthetic gene clusters (BGCs). For most known gene clusters, the produced metabolite is unknown and various approaches have been explored for the prediction of SM scaffolds [2]. However, for most known SMs, the encoding BGC has not yet been discovered.

A first general approach in connecting SMs and BGCs was made with our SeMPI v1 web server [3]. The server combined predictions of modular polyketides (PKs) with a screening of putative matches in the StreptomeDB [4], a database dedicated to natural products produced by streptomycetes. In SeMPI v2, this approach has now been extended by including nonribosomal peptides (NRPs) to the prediction scope. Additionally, the database screening was increased by seven publicly available natural compound-related libraries, allowing the screening of more than 190,000 compounds. The new libraries include the NANPDB [5], ChEBI [6], Drugbank [7], Analyticon [8], Molport [9], and Norine [10] database.

Whereas SeMPI v1 was dependent on antiSMASH v3 [11] as the BGC prediction backend, SeMPI v2 uses an entirely independent prediction approach. For the new pipeline, various improvements were implemented. The profile hidden Markov models (profile-HMMs) used to detect biosynthetic domains were benchmarked to determine sensitive detection thresholds. The biosynthetic modules of known BGCs were investigated to improve the building block generation. Additional methylation patterns were implemented for PK and NRP building blocks. A novel algorithm was designed to determine the possible module order for clusters that do not follow the co-linearity principle [12]. Polyketide synthase (PKS) acetyltransferase (AT)-domain and nonribosomal peptide synthetase (NRPS) adenylation (A)-domain substrate specificities are predicted using random forest (RF) models. To increase the range of A-domain specificity predictions, a novel, very large training dataset of 2145 sequences was generated. To predict postsynthetic modifications (PSMs), correlating domains annotated in the Pfam database v32 [13] were extracted and used to build regression models. The database screening was improved by extending the path-based algorithm used in SeMPI v1 to a maximum common substructure-based (MCS) algorithm.

The prediction and ranking performance was benchmarked using 559 PKS and NRPS BGCs with known substrates from the MIBiG database v2 [14]. Compared to antiSMASH v5, SeMPI v2 predictions achieved better overall performance. Benchmarking of the ranking allowed the identification of the correct SM among the Top 10 of the 559 SMs in almost 50% of all cases.

The web server provides detailed information about the cluster, including a genome browser which allows visual investigation of the cluster. The predicted SMs can be modified and extended using a molecular workbench before submission to the database screening. Custom molecule libraries can be used to extend the default natural compound screening database. The SeMPI v2 web server is available at http://sempi.pharmazie.uni-freiburg.de.

## 2. Results

### 2.1. Benchmark of the PKS and NRPS Profile-HMMs

BGC detection relies on the correct identification of domains involved in SM biosynthesis. Therefore, the profile-HMMs used to detect biosynthetic domains were benchmarked and sensitive detection thresholds were determined. The cross-validation results and detection thresholds (domT) of the profile-HMMs are shown in Table 1. Most profiles show very high F-scores above 0.95. Only two domains reached F-scores below 0.9: the bACP domain (0.59) and the DHt domain (0.88).

The precision for the bACP domains reached only 0.42, meaning that many domains are falsely classified as bACP. A detailed investigation of the cross-validation results showed that only the ACP domains were classified as bACP. In total, 215 ACP domains were classified as bACP, as opposed to 155 truly detected bACP domains. The recall of 0.8 of the DHt domain shows that, in rare cases, this domain is classified wrongly. The cross-validation results revealed that the tAT_d can be detected as a DHt domain. This happened in 21 instances, as opposed to 91 truly detected DHt domains. In one case, the DH2 domain was detected as a DHt domain.

### 2.2. PKS and NRPS Domain Architecture

**Figure 1:**
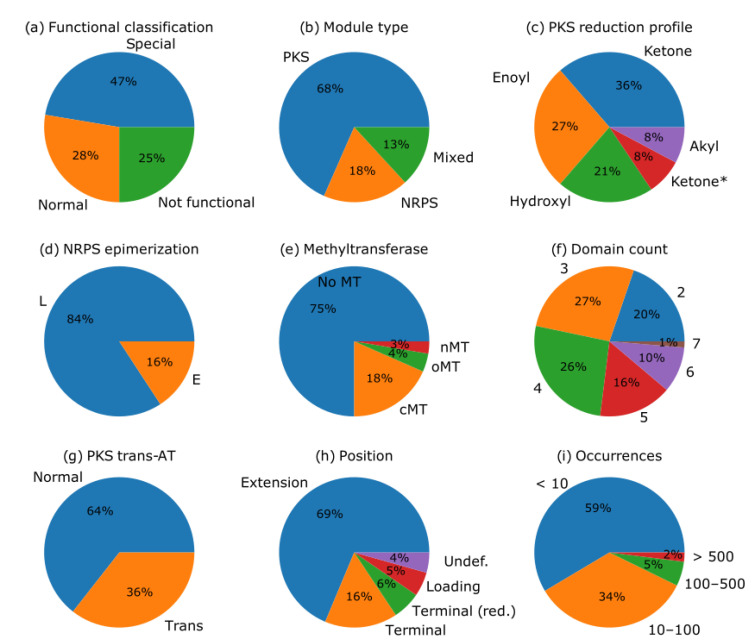
Summary of MIBiG module arrangements which occur at least twice in the MIBiG v2 database. The summary includes: functional classification (**a**) as explained in Section 4.2; module type (**b**), where mixed refers to modules which are composed of both PKS and NRPS domains (e.g., C-A-ACP); reduction profile of PKS modules (**c**), where the special reduction profile ketone * refers to modules in which reductive domains are present but the reduction cannot be performed because the KR module is missing (e.g., KS-AT-DH-ACP); epimerization of the NRPS module (**d**), where L refers to the L-configuration of the substrate and E refers to the R-configuration of the substrate; the presence of MTs (**e**); number of domains in the modules (**f**); classification of PKS modules into normal and trans-AT acting modules (**g**); position in the BGC (**h**); and number of overall occurrences in the database (**i**).

To improve the existing PKS domain patterns for module detection and extend the detection scope to NRPS and PKS-NRPS mixed modules, the BGCs in the MIBIG v2 were observed and categorized. A summary of the extracted modules is shown in Figure 1 and the 20 most common modules are listed in Table 2. A complete list with all 212 module arrangements is shown in Supplementary Materials Table S1.

### 2.3. Adenylation and Acyltransferase Substrate Specificity

#### 2.3.1. Adenylation Domain Dataset

**Figure 2:**
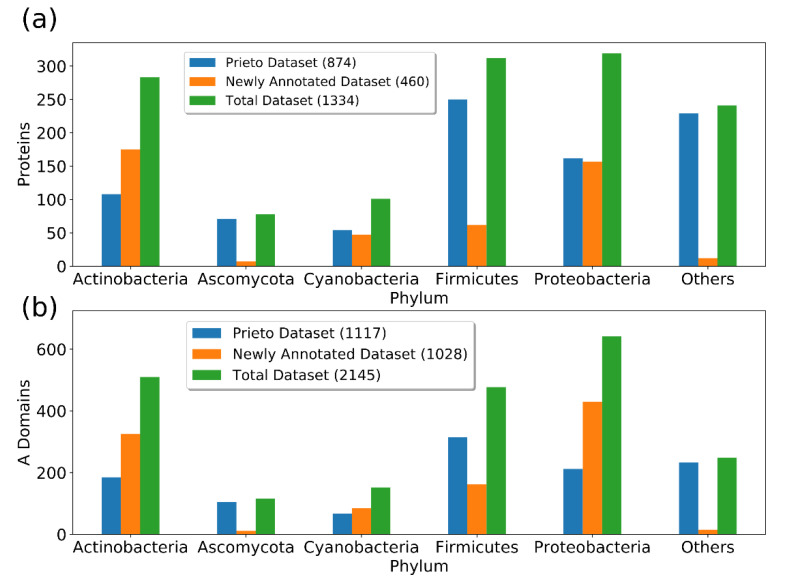
Phylogenetic composition of the A-domain training dataset. The composition is shown for each protein (**a**) and all the A-domains (**b**). Almost all fungi sequences can be attributed to the phylum of ascomycota. The division of others comprises domains where no taxonomy could be found or small groups, such as *streptophyta* (No. 6)*, candidatus tectomicrobia* (No. 5), and *arthropoda* (No. 3).

In order to improve the performance of the A-domain specificity prediction models, a novel, very large training dataset was generated. Overall, 1028 new A-domains could be collected, leading to 2145 sequences altogether. An overview of the phylogenetic composition of the dataset is shown in Figure 2. The largest amount of A-domain sequences could be collected for proteobacteria (642) and actinobacteria (510). The dataset for actinobacteria, cyanobacteria, and proteobacteria could be more than doubled. The fungus dataset, represented mainly by ascomycota, could only be increased by 11 domains. The complete domain list is provided in Supplementary Table S2.

#### 2.3.2. Random Forest Parameter Grid Search

Different parameters for the RF models were evaluated using a grid search based on the cross-validation described in Section 4.4.2. The best parameters for the RF models for both domain types are shown in Table 3.

#### 2.3.3. Classifier Performance

The RF models with the best parameters determined in Section 2.3.2 were benchmarked for individual substrate prediction as well as their overall performance. The individual performances per substrate class for the AT-domains are shown in Table 4 and for the A-domains in Table 5. As a general trend, it can be observed that the performances improve with the number of training samples. For example, the F1-scores for methylmalonyl and malonly predictions are much higher compared to substrates with fewer samples such as ethylmalonyl and methoxymalonyl. A similar tendency can be observed for the NRPS substrates. However, there are also a few exceptions from this trend. For example, 3,5-dihydroxy-phenyl-glycin, with only 12 training samples, has an F1-score of 1, and phenylalanine, with 122 training samples, can only be predicted with an F1-score of 0.4.

The different metrics for the overall performance of each classifier are shown in Table 6. Macro-averaged scores, which take all classes equally into account, show poorer performance as compared to micro-averaged scores, for PKS and NRPS substrates. The AT-domain classifier shows a very high performance, with an accuracy of 0.95. Even the MCC-score, which is more suited to representing unbalanced classifiers, has a score of 0.91 for the AT-domain classifier. The A-domain classifier only reaches an accuracy of 0.76 (MCC: 0.74), which is still an acceptable performance given the large amount of possible substrates.

### 2.4. Detection of Postsynthetic Modifications

Pfam domains were correlated to specific PSMs and used to build regression models for PSM prediction. Correlating Pfam domains are listed in Table 7. For most domains, the Pfam description matches the synthesized PSM. For example, the involvement of the pyridine nucleotide-disulphide oxidoreductase (Pfam ID: PF07992.13) in disulfide synthesis or the diaminopimelate epimerase (Pfam ID: PF01678.18) in nitro group synthesis can be explained with the function of the specific domains. In a few cases, highly correlating domains could be detected, where the involvement in PSM synthesis is not evident from the description. For example, the role of PF12680.6 and PF00890.23 in spiroketal synthesis or PF06722.11 in nitro group synthesis should be further investigated.

The Pearson correlation coefficient is rather average for all observed domains, with less than 0.67. Hence, most PSMs cannot be predicted based on linear correlation to one specific domain. Therefore, regression models that take multiple domains as features were generated to predict the number of PSMs. The performances of the regression models are shown in Table 8. The models were compared to hypothetical baseline models that compute the R2-score (coefficient of determination) for the case in which no PSM would be predicted for each sample BGC. The regression models could outperform the baseline models in all cases. In particular, the addition of glyco, spiroketal, and 6-Ring-formation PSMs could greatly improve the prediction scope.

### 2.5. Benchmark of the Scaffold Prediction

#### 2.5.1. Similarity of the Predicted Scaffolds to the True Metabolites

SeMPI v2 and antiSMASH v5 were benchmarked based on the Tanimoto similarity of the predicted scaffolds to the true SMs of all MIBiG v2 BGCs. The average (arithmetic mean) Tanimoto similarities for each BGC type are shown in Table 9.

#### 2.5.2. Grid Search for the Best Weights of the Mixed Ranking Score

Different scoring metrics were combined to allow for the best comparison of natural compounds to the predicted scaffolds. The ranking benchmark shown in Section 2.5.3 was used to determine the optimal weight for the mixed ranking score. The best weights and corresponding benchmark performance for each BGC type are shown in Table 10. The parameter configuration which led to the best Top 10 ranking for all BGC types was chosen for the final mixed score.

#### 2.5.3. Metabolite Ranking Database Comparative ranking

The ranking performance of SeMPI v2 and antiSMASH v5 predictions was benchmarked based on the ranking of the MIBiG compounds. The percentages of true SMs ranked into the Top 10 and Top 50 similar compounds are shown in Table 11. The mixed score is based on the grid search performed in Section 2.5.2. Since antiSMASH v5 does not predict PSMs, the mixed score can only be used for SeMPI v2 predictions. The ranking performance of SMs predicted from both pipelines can still be compared when the Tanimoto similarity- or MCS-based ranking is considered.

### 2.6. Web Server Design and Features

**Figure 3:**
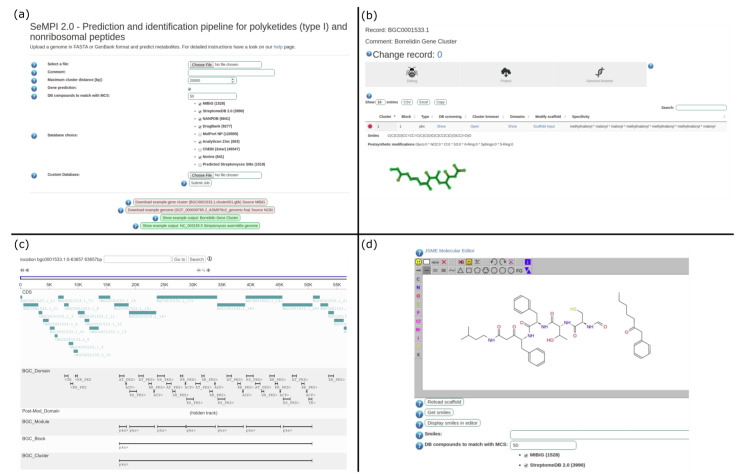
Screenshots of SeMPI v2 web server features. (**a**) The input form allows for the selection of the BGC detection window, the number of natural compounds to compare to the predictions, and the selection of different public and local databases for the screening. (**b**) The results page provides detailed information about the predicted scaffold as well as links to further analysis resources. (**c**) The genome browser allows the observation of all biosynthetic relevant domains, genes, and clusters in the surrounding genome. (**d**) The molecular workbench enables expert users to submit custom molecular queries to the database screening.

The SeMPI v2 web server is available at http://sempi.pharmazie.uni-freiburg.de and has processed hundreds of genomic input files without error (except for wrong input files). The processed files include 200 complete streptomyces genomes and all PKS, NRPS, and PKS-NRPS hybrid BGCs from the MIBiG, which were added to the server as a database feature. The server has been running continuously since 26 March 2019, without incidents. A detailed description of its features and usage is provided at the help page of the web server. A selection of screenshots is shown in Figure 3.

## 3. Discussion

### 3.1. Profile-HMMs and Detection Thresholds

The overall high F-scores above 0.95 indicate that the MIBiG-derived pHMMs are appropriate tools for BGC detection. Only the ACP and bACP domains are wrongly classified in some cases, but since the role of ACP and bACP is identical for the SeMPI v2 module detection algorithm, the misclassification does not pose a problem for the overall scaffold generation. The DHt profile-HMM could be improved, but the detection error is still rather low and the domain is only seldom found in a BGC. The MIBiG v2 contains considerably less DHt domains (133) compared to DH (1469) and DH2 (414) domains.

The detection thresholds (domT) were derived from the minimum scores needed to detect the true domain in the MIBiG. It seemed reasonable to use the curated MIBiG as a reference for domain detection. Nevertheless, the threshold should be frequently updated with newly added BGCs in the database. The only core profile-HMM which is not derived from this analysis is the KSQ profile since it was not separately labeled in the GenBank annotation of the MIBIG. The profile-HMM was directly taken from the antiSMASH v5 pipeline. The same threshold as for the KS domain was used.

### 3.2. PKS and NRPS Domain Architecture

To assign biosynthetic module arrangements with corresponding SM building blocks, a detailed investigation of the extracted modules was performed.

#### 3.2.1. The Position and Role of the Methyltransferases in PKS

A detailed investigation of the carbon methyltransferase (cMT) containing modules revealed that the cMT domain can be found primarily in three possible arrangements in a PKS module. First, the cMT can be positioned between the reduction domains. For most modules containing reduction domains, the cMT was integrated at a conserved position (e.g., BGC0001000 [KS-AT-cMT-KR-ACP], BGC0001136 [KS-AT-DH-cMT-KR-ACP], and BGC0001001 [KS-AT-DH-cMT-ER-KR-ACP]). Second, if there were no reduction domains present, the cMT was located between the AT and the ACP/PCP domains (e.g., BGC0000017 [KS-AT-cMT-ACP] and BGC0001203 [KS-AT-cMT-PCP]). Third, surprisingly, trans-AT associated modules showed a different cMT arrangement as opposed to the normal (cis-AT) modules: the cMT was positioned between the reductive domains and the ACP domain (e.g., BGC0001071 [KS-tATd-DH-KR-cMT-ACP], BGC0000177 [KS-tATd-KR-cMT-ACP], BGC0001470 [KS-tATd-KR-cMT-bACP], and BGC0000186 [KS-tATd-DH-KR-cMT-bACP]).

Only one example of a trans-AT module with three reductive domains is described in the MIBiG v2 (BGC0001106). Since this module does not include a cMT domain, the correct cMT position for this arrangement could not be inferred. The characteristic cMT position could also be observed for putative trans-AT modules, where the trans-AT docking domain could not be detected (e.g., BGC0000177 [KS-DH-KR-cMT-ACP]), which could be a further marker for trans-AT detection.

The module arrangements described above were integrated into the module detection algorithm. The corresponding molecular scaffolds were derived by the addition of a methyl at the α-position of the building block. This could be observed for cis-AT [17,18,19] as well as trans-AT [18,20] modules.

A special case is represented by the methylmalonyl substrate, where the building block is already α-methylated. In this case, a second α-methylation is also possible (e.g., in the case of Kirromycin [21]). However, the reduction to the double bond or alkyl is chemically not possible for this scaffold. This is confirmed by the BGC encoding for epothilone (BGC0000989 [22]). In the eighth module, the arrangement of DH-MT-KR leads to an α-methylation of the methylmalonyl substrate, but the reductive domains are inactive.

**Figure 4:**
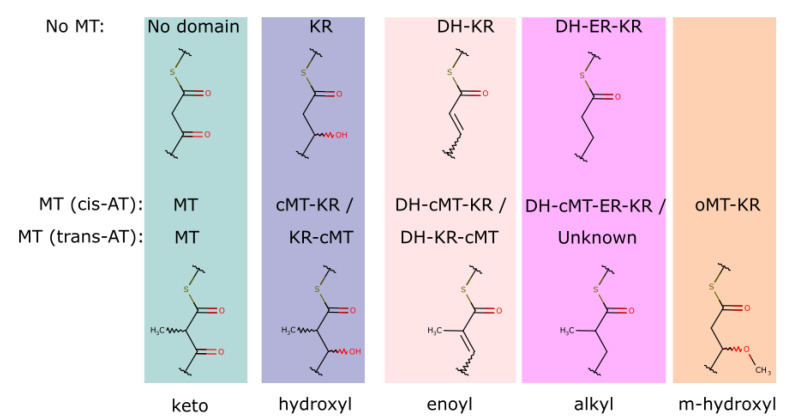
Reduction and methyltransferase domain combinations for described PKS modules. The resulting building block modifications are depicted below the domain combination. The methyltransferase position for cis and trans-AT modules are shown. Each domain combination (X) is encapsulated such as KS-(AT)-X-ACP. The figure is based on the article by Nguyen et al. [18].

The oxygen methyltransferase (oMT) could be found predominantly (16 times) in modules that only contain a KR domain (e.g., BGC0000976 [KS-AT-oMT-KR-ACP]). In those cases, the keto-group in β-position is first reduced to a hydroxyl group and subsequently methylated. The oMT domain could also be observed in modules lacking reduction domains. Trans-AT modules (e.g., BGC0001110), as well as cis-AT modules (e.g., BGC0000982) without reduction modules, have been observed harboring an oMT domain. Although biosynthesis routes for BGCs with these module types have been proposed (cis-AT [23] and trans-AT [11]), the function of the oMT domain in these modules is too specialized and the proposed biosynthesis routes are only hypothetically described in publications. Therefore, a general rule for the transformation performed by these module types was not included in the module detection algorithm. A summary of all PKS module arrangements detected by SeMPI v2 and corresponding building blocks is shown in Figure 4.

#### 3.2.2. The Position and Role of the Methyltransferases in NRPS

The most prominent MT found in NRPS modules is the N-methylating type. The arrangement C-A-nMT-PCP can be observed 120 times in the MIBiG, being the most commonly occurring methylation. The nMT is located between the A and PCP domain in all observed cases, e.g., BGC0001971 (C-A-nMT-PCP), BGC0000461 (C-A-nMT-PCP-TE), BGC0000384 (A-nMT-PCP), and BGC0000326 (C-A-nMT-PCP-E). The nMT domain usually leads to methylation of the nitrogen in the peptide bond of the inserted building block (e.g., retimycin and rakicidin A [24]).

In very rare cases, a C-methyltransferase (cMT) is also observed (C-A-cMT-PCP-TE). This cMT is responsible for the methylation of the α-carbon of a thioproline building block in thiazostatin-like metabolites (e.g., BGC0001014, BGC0000324, and BGC0001801). This reaction is too specific to infer a general biosynthetic rule.

#### 3.2.3. NRPS-PKS Hybrid Modules

A detailed investigation of PKS-NRPS mixed modules showed that, in all cases, the ACP/PCP domain led to the classification as a mixed module (e.g., C-A-ACP or KS-AT-KR-PCP). These domains are structurally related and share similar tasks, namely the transport of the building blocks. Therefore, it is probable that mixed forms exist which cannot be reasonably distinguished with pHMMs. In some cases, they might be wrongly detected (e.g., BGC0001699). In other cases, especially in PKS-NRPS mixed clusters, the ACP/PCP domain could represent the link between the PKS and the NRPS part of the cluster (e.g., BGC0000459). In regard to the building block generation, this domain swap can be disregarded, since the substrate carrying domains do not lead to modifications of the building block.

#### 3.2.4. Scaffold Generation

**Figure 5:**
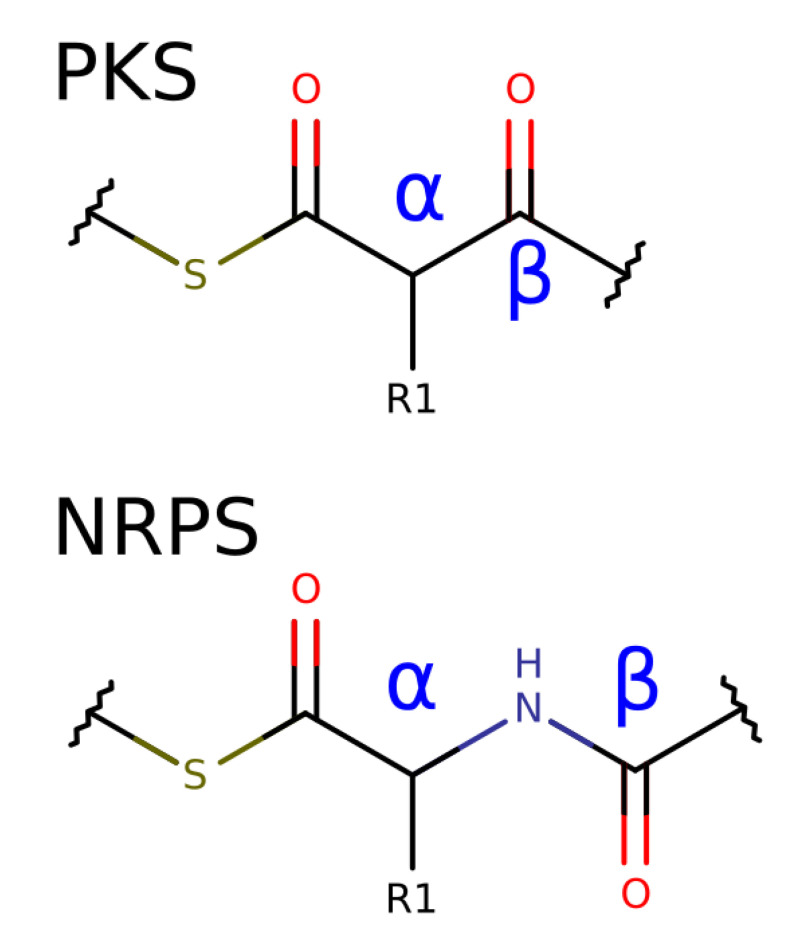
Nomenclature for PK and NRP building blocks. R1 in alpha position is defined by the substrate choice of the AT- or A-domain. The α-position of PK substrates can be further modified co-synthetically by C-methyltransferases. The stereo configuration of the α-carbon of NRP substrates is defined by the presence of an E domain. The β-position of PK substrates can be further reduced. For NRP substrates, only the nitrogen atom, also referred to as β-position, can be methylated.

Based on the module analysis, detection rules for module arrangements with known substrates were derived. For each module, a building block is generated using SMILES. The building block represents the attachment of two carbon atoms to the growing metabolite in the case of PKs. The positions are labeled as described in the article by Keatinge-Clay [25] and are shown in Figure 5. NRP building blocks add a peptide bond and a carbon atom to the growing chain. Analogous to PK, the NRP positions are referred to as β for the peptide bond and α for the added carbon with the side chain.

The PKS reduction pattern and putative methylation are created as described in Figure 4. For the building block generation, it is important to consider that the reductive domains act on the previous building block (not the one activated in the observed module) and therefore this modification needs to be assigned accordingly. There are two possible NRP modifications. First, the stereo configuration of the α-atom can be modified depending on the presence of an epimerization domain. Second, the nitrogen of the peptide bond can be methylated.

PKS loading models are defined by the presence of a KSQ domain or the absence of a KS domain. NRPS loading modules are defined by the absence of a condensation domain. Building blocks of loading modules are missing the carbon atom in β-position. Terminal modules for both cluster types are defined by the presence of a TE or TD domain. Additional to the β- and α-position, these modules add a carboxyl group (TE) or a hydroxyl group (TD) to the building block.

**Figure 6:**
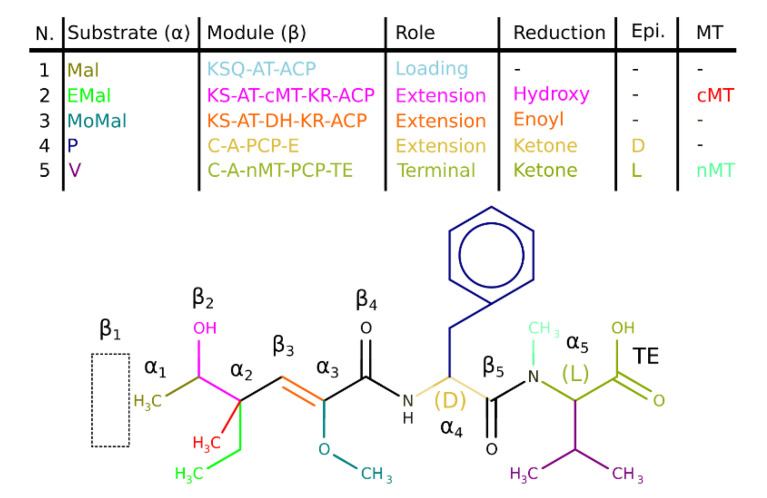
Scaffold generation for a hypothetical mixed cluster. The NRP positions are marked α for the N-C-rest and β for the ketone following the PK nomenclature. The substrate specificity leads to a modification in the α-position. The module arrangement results in a modification in the β-position. Each parameter and corresponding modification is color-coded. Loading modules do not add a carbon atom in the β-position, marked with a dashed box (β_1_). Terminal modules add an additional carboxyl (TE) or hydroxyl (TD) substructure to the scaffold. Epi. refers to the epimerization of the NRP substrates, resulting in L- or D-forms.

For each complete block, the building blocks are joined together to generate the final metabolite. An example of the scaffold generation for a hypothetical mixed cluster is shown in Figure 6.

### 3.3. PKS and NRPS Module Order

The correct order of modules in clusters that deviate from the co-linearity principle is difficult to predict. In some cases, the correct order can be derived by arranging starting modules at the beginning of a block and terminal modules at the end. However, for clusters composed of multiple disjointed modules, this simple approach does not suffice.

Although docking domains for PKS allow one to infer the order of modules in some cases, in many clusters, these domains are missing or cannot be identified with the existing profile-HMMs. In the study by Yadav et al. [26], 17 clusters were used to demonstrate the possibility of predicting the module order based on docking domains. A larger study has not been performed yet. Moreover, additional structurally different docking domains (class 2) have been described [27], further complicating the prediction of PKS module order. To the best of our knowledge, for NRPS and hybrid BGCs, no such study has been performed yet.

Since clear ab-initio rules are missing, SeMPI v2 infers the order of modules by comparing the overall domain architectures with known clusters. This approach could lead to correct module compositions for various clusters including nigericin (BGC0000114), incednine (BGC0000078), fluvirucin B2 (BGC000157), and even meilingmycin (BGC0000093)—which is composed of a very complex composition of six disjointed blocks. A final assessment of the method would require a large selection of disjointed BGCs, where the correct order is derived from the literature. Since such a collection is not yet available, the ordering algorithm was measured indirectly based on the quality of the predicted scaffolds (see Section 2.5.1 and Section 2.5.3).

### 3.4. Adenylation and Acyltransferase Substrate Specificity

In the last decade, various methods have been proposed for the prediction of the substrate specificity of AT- [28,29,30,31,32] and A-domains [30,31,33,34,35,36]. For each study, different datasets with known substrates have been collected and used to implement a prediction algorithm or train machine learning models. Although the studies tried to compare their predictive accuracy against each other, in most cases, this comparison is of little significance, since the used datasets are not identical. Different cross-validation methodologies and classification metrics further hinder the comparability of substrate classification models. Despite these difficulties, it could be observed that most substrate prediction tools reach rather similar performances, especially concerning the most common substrates.

For AT-domain substrate predictions with a rather small substrate choice, the accuracy reaches more than 90%. For example, Khayatt et al. could predict 163 domains with 95% [35] accuracy and Minowa et al. reached an accuracy of 93% for 471 domains [37]. A-domain predictions which are more challenging—due to their large substrate range—reached F1-scores (micro or macro) between 0.7 and 0.8. The latest study, SANDPUMA [36], could demonstrate that various methods achieve this performance. However, it should be mentioned that the demonstrated ensemble method is slightly better than the individual classifiers.

The RF models used for substrate prediction in SeMPI v2 yielded comparable performances with the aforementioned state-of-the-art tools. The AT substrates could be predicted with an accuracy of 95% and the A-domain specificity classification reached a micro F1-score of 0.76.

The observation of a performance peak in substrate predictability allows the assumption that further improvements should be focused on the training dataset, rather than on the used algorithm. It can be hypothesized that various algorithm-independent parameters are responsible for the difficulty of substrate specificity classification. First, substrate variability hinders the predictability of one outcome class [38]. Second, substrate classes with few known instances are difficult to predict. Finally, since the training datasets are manually collected, wrongly assigned specificities can further impair the predictive power.

Therefore, a strong focus was placed on increasing the A-domain training dataset for the SeMPI v2 classifier. The dataset collected by Prieto et al. [34] comprised the largest collection of annotated A-domain sequences (1598). Surprisingly, even the dataset used for the SANDPUMA algorithm—which is composed of the dataset collected by Khayatt et al. (494) and newly annotated sequences (434)—was smaller than the Prieto dataset (928). Consequently, we decided to use the Prieto dataset as a basis and added manually annotated sequences, which allowed us to generate the largest dataset of annotated A-domain sequences of overall 2145 samples.

### 3.5. Detection of PSMs

PSMs are more challenging to predict than the rather straightforward linear assembly lines of the PKS and NRPS core modules. This is partly because the involved domains are rather arbitrarily located in proximity to the BGC. However, the main reason can be attributed to missing learning data. Although hundreds of BGCs and their SMs are stored in the MIBiG, only a few instances of each PSM can be found—the only exception being carbohydrate scaffolds, where various examples are known, but the type and number of glycosides vary greatly between clusters.

Therefore, the scope of PSMs in SeMPI v2 is mainly targeted by the subsequent database screening. Nevertheless, for some PSMs where at least a few instances are described, a prediction was attempted. Eventually, the addition of PSMs into the screening process can bring researchers closer to the true product of a BGC. The correlation analysis yielded various Pfam domains that were positively correlated to a given PSM. The regression models built based on these domains could predict the number of PSMs for a cluster with a high R^2^-score.

For carbohydrate scaffolds, only the number of glycosides is predicted since the prediction of the glycoside type depends on complex domain arrangements. To the best of our knowledge, only PRISM v3 [39] provides a combinatorial approach for sugar prediction, although a detailed benchmark of the method is missing.

### 3.6. Benchmark

#### 3.6.1. Similarity of the Prediction to the True Secondary Metabolite

The comparison of the predicted scaffolds with the true metabolite shows that the predictions made by SeMPI v2 are slightly better compared to the antiSMASH v5 predictions. The best predictions are made for NRPS products (mean Tanimoto similarity: 0.57), which shows that the A-domain specificity classification of SeMPI v2 can compete with the SANDPUMA algorithm implemented in antiSMASH v5.

The rather average Tanimoto similarity between prediction and true SM for both tools can be seen as a general indicator of how much structural information is still missing in the prediction scope. Some of this structural information gap can potentially be completed by comparison of the prediction with similar, already described compounds.

#### 3.6.2. Ranking of the True Metabolite

For the NRPS products, the MCS-based ranking yielded rather similar performance for antiSMASH v5 (Top 10: 45.9%) and SeMPI (Top 10: 45.1%) predictions. However, for the PK dataset, the MCS-based scoring performed much better for SeMPI v2 predictions (Top 10: 36.0%) as compared to antiSMASH v5 (Top 10: 22.5%) predictions. Since the similarity benchmark did not show such a discrepancy, this observation was further investigated.

The careful observation of various PK predictions showed that antiSMASH v5 predictions have an offset concerning the reduction patterns of PK products. Furthermore, in the observed scaffolds, the double bonds were assigned at the wrong position. A demonstration based on the abyssomicin BGC is shown in Supplementary Figure S1. This wrong assignment led to false matching in the MCS algorithm for antiSMASH v5 predictions. This observation could demonstrate the advantage of the MCS-based ranking as opposed to Tanimoto similarity-based ranking. To further increase the ranking power, the three ranking scores were combined to a mixed score, which yielded the overall best possible ranking. This mixed score ranked 47% of the true SM into the Top 10.

The NRPS–PKS hybrid BGCs led to the poorest rankings in the benchmark. This is not surprising, since NRPS–PKS mixed BGCs can lead to very complex scaffolds, which are difficult to match with the linear predictions. Nevertheless, 40% of the mixed products could be ranked in the Top 10. Considering the Top 50 ranked compounds, which is the default output on the web server, 66% of the true SMs were matched.

The ranking benchmark demonstrates that researchers aiming to identify BGC products can gain significant support by using the SeMPI v2 database screening approach.

## 4. Materials and Methods

### 4.1. PKS and NRPS Profile-HMMs

The widely used domain annotations from the MIBIG database v2 [14] were taken as a basis for constructing new profile-HMMs for the SeMPI v2 pipeline. The domain names were based on the commonly used antiSMASH [4,11,16,40,41] nomenclature.

To evaluate the detection sensitivity of the created profiles, a benchmark was designed where the profiles were 5-fold cross-validated. Precision, recall, F1-score, and the support for each profile are reported. Additionally, the detection threshold (domain-specific e-value as reported by the HMMER software [15,42], referred to as domT) was adjusted for each profile-HMM. The minimum threshold required for the detection of the correct domains in the MIBIG v2 was used for each profile-HMM in the SeMPI v2 pipeline.

### 4.2. PKS and NRPS Module Architecture

Domains that are jointly responsible for the incorporation and co-modification of a new building block in the synthesis of a SM are defined as modules. To systematically investigate known module architectures and their associated building blocks, NRPS, PKS, and PKS–NRPS hybrid modules were extracted from the MIBIG v2 database. Specific properties which allowed us to examine and categorize the modules were computed: the number of occurrences of the module in the MIBiG, the MIBiG accession IDs, the order of the domains, the occurrence of methylation domains, the reduction or epimerization profile, and the possibility of a trans-acting AT.

The modules were categorized into three groups. First, the normal modules comprised domain sets for which complete building blocks could be derived. These modules contained either an A-domain or an AT-domain, allowing classification of the substrate and co-synthetic modifications of the building block. For the PKS system, modifications comprised the reduction profile as well as possible methylation of the substrate. For the NRPS system, the stereo-configuration of the α-carbon was determined as well as possible methylations. Additionally, for releasing modules, the possible reduction of the carbonyl group was determined.

Second, the special modules were defined as modules for which building blocks could only be partly defined. For these modules, either the substrate specificity could not be predicted (e.g., trans-AT) or the co-synthetic modifications could not be inferred (e.g., PKS modules with an unusual reduction domain arrangement, such as KS-AT-DH-ACP).

Third, the non-functional modules comprised all domain combinations which did not allow us to determine a possible building block, because crucial domains for the function of the module were missing (e.g., DH-ACP, C-PCP, KR-ACP).

Based on the functional and special modules, patterns were derived, which can be used to screen novel BGCs for known modules.

### 4.3. PKS and NRPS Module Order

An algorithm was designed which allows us to score possible module combinations in a BGC based on known BGC arrangements.

#### 4.3.1. Database of Clusters with Known Module Order

The MIBiG v2 BGCs modules were combined into blocks. Each block represented a unit in which the modules were arranged in succession. Only modules that were located directly next to each other on the same DNA strand were combined into blocks. The blocks were split up if a terminal module (harboring a TE or TD domain) was not located at the end position of the block.

Those BGCs in the MIBiG v2 database, where exactly one block could be defined, were taken as reference BGCs. The reference database comprised 252 BGCs.

#### 4.3.2. Module Order Scoring for Novel Clusters

For a novel BGC, the modules are ordered into blocks. For all blocks in a BGC, an all-vs.-all matrix is computed. For each pair in the matrix, an artificial domain set is generated, representing hypothetical combined blocks. Only reasonable block combinations are considered. For example, starting domains can only be at the beginning of a block pair and terminal domains can only be at the end of a pair. The generated domain combinations are aligned with the reference BGCs and scored.

**Figure 7:**
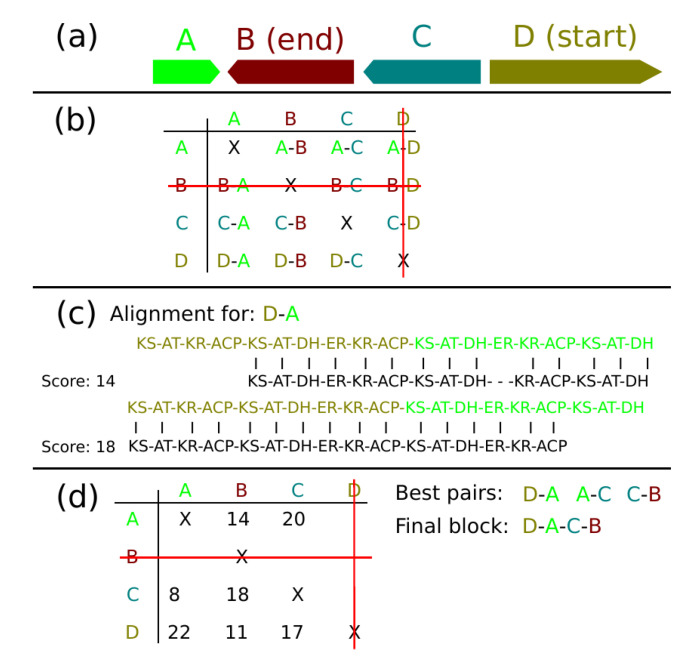
Demonstration of the module order algorithm. (**a**) Example blocks which cannot be combined since they are on different strand sides or far apart in the cluster. (**b**) An all-vs.-all matrix which generates hypothetical combined block pairs. Each block can be either at the beginning or the end of the newly generated block. Unreasonable combinations are excluded, marked with a red line. (**c**) A hypothetical example of 2 alignments performed for the D-A block combination. Each combination is scored with 252 reference BGCs. (**d**) Based on the score assigned to each block pair, the best scoring pairs are extracted and combined to the final block.

The alignment is performed using the Smith-Waterman algorithm [43] for local alignments. The parameters are matched domains yield 1 point, unmatched domains cost 1 point, gap opening costs 2 points, and gap extension costs 1 point. The block pairs which yield the highest scores are combined into the final blocks. If not all blocks can be combined (e.g., if two terminal domains are present), the blocks are left disconnected. The module order algorithm is demonstrated in Figure 7.

### 4.4. Adenylation and Acyltransferase Substrate Specificity

#### 4.4.1. Sequence Datasets

Sequences of PKS AT-domains with known substrate specificity were taken as described in SeMPI v1 [3]. The initial set of annotated NRPS A-domain sequences was taken from Prieto et al. [34]. This dataset comprised 1598 annotated A-domains. The dataset was used to generate a profile-HMM [15,42,44]. The profile-HMM was used to screen the entire MIBiG v2 [14] and collect a novel set of A-domain sequences. The sequences collected by Prieto et al. and the newly collected sequences were aligned using MAFFT (for multiple alignment using fast Fourier transform) [45]. Duplicated sequences with an identity of 100% were removed. Substrate specificities for the new sequences were inferred from the scientific literature of described metabolites.

For both sequence datasets, only sequences where at least 10 known specificities could be assigned were used. The A-domain dataset was further trimmed to only include unambiguous specificities. The final datasets comprised 500 AT-domains and 2145 A-domains. The A-domains were furthermore phylogenetically categorized. Therefore, the taxonomy for each sequence was derived from UniProt [46].

#### 4.4.2. Specificity Prediction

**Figure 8:**
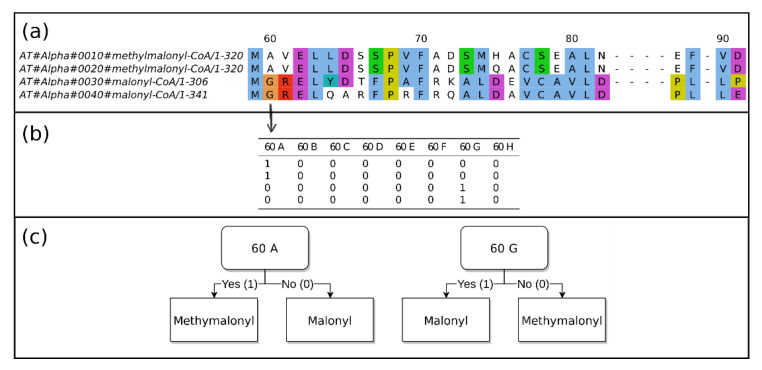
Explanation of the sequence encoding scheme. (**a**) The annotated sequences are aligned. (**b**) Each position in the alignment is encoded in a binary feature using one-hot encoding. (**c**) The RF models are trained on the encoded arrays. A very simplified decision is demonstrated. On the live system, many more positions are taken into consideration.

The sequences were used to train random forest (RF) classifiers. The sequences were aligned using MAFFT. The alignments were transformed into an array that could be used as a feature set to train RF models. Each position in the alignment can be interpreted as a categorical feature. The RF models were implemented using scikit-learn [47]. The scikit-learn models require binary features; hence, the categorical feature space was transformed into binary features using one-hot encoding (also referred to as dummy encoding). The encoding scheme and model building are visualized in Figure 8.

To determine the best parameters for the RF classifiers, an extensive parameter grid search was performed. The parameters are listed in Table 12. All parameter combinations resulted in overall 2160 configurations. For each parameter combination, a model was built and evaluated using 10-fold cross-validation. The model performance was evaluated using the micro F1-score. For the models with the best configuration, the performance for individual substrates was further evaluated. To allow comparison with other methods, various metrics for the cross-validation were computed: accuracy, precision, recall, F1-score, area under the receiver operating characteristic curve (ROC-AUC), error rate, and Matthews correlation coefficient (MCC).

Precision, recall, and F1-score were computed individually for each class. To evaluate the power of the entire model, the metrics were also averaged using micro-, macro-, and weighted-averaging. Similarly, micro- and macro-AUC scores were computed.

### 4.5. Detection of Postsynthetic Modifications

The GenBank annotation of the MIBIG database v2 was used to extract the number of non-overlapping Pfam domains present in each BGC. The scaffolds of the produced SMs were screened for putative PSMs using the substructure search as implemented in RDKit [49]. SMARTS patterns used to match the PSMs are shown in Table 13.

The number of Pfam domains per BGC was correlated with the number of substructures in the related metabolites using the Pearson correlation coefficient. The positively correlating domains were further investigated to derive their function in the PSM. The investigation was based on the Pfam description. For example, domains that correlated with sugar scaffolds were examined for sugar biosynthesis association, such as glycosyltransferase activity. The selected domains were used to build logistic regression models with scikit-learn. The models were evaluated with 5-fold cross-validation. The performance was measured using the R^2^-score (coefficient of determination) [50]. As a baseline, the R^2^-score was also computed for a hypothetical model, assuming no PSMs are predicted at all. The models which performed better than the baseline were incorporated into the prediction pipeline. The predicted PSMs were added as additional features to the predicted scaffold.

### 4.6. Natural Product Database Screening

#### 4.6.1. Natural Compound Databases

SMILES of various open-source databases with a focus on natural compounds were collected. The StreptomeDB v2.0 [4], NANPDB [5], ChEBI [6], and Drugbank [7] molecules were retrieved via their web interface in SDF format. ChEBI contains manually curated and automatically collected molecules. Only the manually curated ones were taken. The MIBiG v2 molecules were extracted from JSON files downloaded via the web interface. Molport molecules were retrieved from the Molport FTP server [9]. Only those molecules which were marked as natural products (NPs) in the database were taken. Analyticon [8] molecules were retrieved via their ZINC [51] deposit. Norine [10,52,53] peptides were downloaded via the REST API of the web interface.

Furthermore, a collection of streptomyces genomes were processed using SeMPI v2, and the results were stored as a database feature on the web server. The predicted BGC products were also included as a possible natural compound screening option. This option allows one to detect the putative BGC for a given streptomyces natural product. The streptomyces genomes included all complete genomes associated with compounds stored in the StreptomeDB v3 [54]. Whereas the database screening allows for the detection of putative SMs for a submitted BGC, the addition of preprocessed BGCs also enables reverse screening, thus allowing one to connect SMs with their putative BGCs. A summary of the databases is shown in Table 14.

The molecules were parsed using RDKit and incorporated into a PostgreSQL [55] database. Duplicated SMILES were removed from the database. The SMILES were converted into circular Morgan fingerprints (radius: 3) using the RDKit database cartridge. The fingerprints were indexed using the Generalized Search Tree (GiST) [56] algorithm.

#### 4.6.2. Predicted Scaffold Scoring

**Figure 9:**
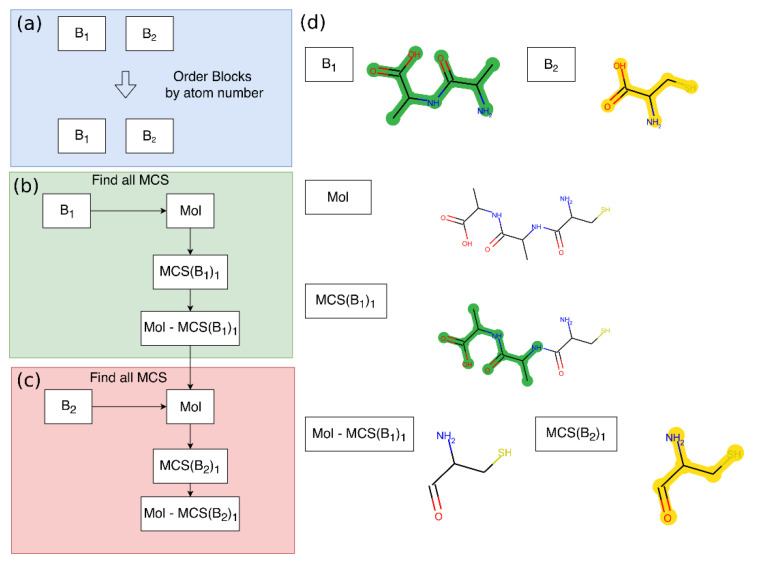
Example demonstration of the extended MCS algorithm. To simplify the example, only two building blocks are used; the algorithm can potentially scale up to 10 building blocks. (**a**) Initially, the building blocks are ordered by their number of atoms. The matches of the biggest building blocks are most meaningful. (**b**) All MCSs of the first block (B1) with the target molecule (Mol) are computed. The example shows only one MCS, but some molecules (especially ring systems) can have large numbers of MCS. The number of MCS to find for each B1 in a target molecule (Mol) is limited to 20. (**c**) A new molecule is created for each MCS, where the MCS is removed from the scaffold. This new molecule is then submitted to a new MCS search with the next building block (B2). (**d**) A visual example of the scoring algorithm based on two predicted scaffold fragments. First, the dipeptide (B1) is scored; then, the second molecule (B2) is scored on the remaining part of the target molecule (Mol).

The database set-up achieves a Tanimoto similarity search of the predicted scaffolds with more than 180,000 molecules in less than a second. The Tanimoto similarity search allows the selection of a group of closely related scaffolds to the query prediction. However, especially repeating patterns such as PK and NRP scaffolds do not allow for good scaffold comparison using molecular fingerprint-based algorithms. Therefore, an additional most common substructure (MCS)-based scoring algorithm was implemented. The MCS algorithm scores the predicted molecules with the compounds in the database, by detection of a MCS. The score is computed by dividing each matching atom and bond in the predicted molecule and the target molecule by the total number of atoms and bonds. Because the MCS algorithm is very time-consuming, it is by default only performed on a preselection of 50 compounds from the similarity search. The RDKit implementation only allows the generation of the MCS between one query and one target molecule. To allow for the matching of incomplete BGCs, the algorithm was extended to allow for the matching of multiple query molecules (e.g., multiple disconnected building blocks) in the natural compound targets. The extended MCS scoring algorithm is demonstrated in Figure 9.

A third scoring algorithm was implemented to cover the influence of PSMs on the ranking of the metabolites. This scoring function performs a substructure search using RDKit for the predicted PSMs on the preselected natural compounds. The match between the PSMs in the predicted scaffold and the natural compound is computed using the Bray–Curtis similarity [57]. The Bray-Curtis similarity is defined as:

BC = ∑|u_i_ − v_i_|/∑|u_i_+v_i_|, (1)

where u is the array of PSMs found in the predicted compound and v is the array of PSMs found in the natural compound from the database. This similarity score was chosen since the interpretation is rather intuitive: if both molecules have the same number of PSMs, the score is 1; the greater the distinction, the closer the score is to 0.

The scores are used to rank the natural products to present a selection of possible complete SMs that might be produced by the observed BGC. For the final ranking, a mixed score was designed where the individual scores are added with different weights:

Mixed-score (a,b,c) = a × Similarity-score + b × MCS-score + c × PSM-score.(2)

To determine the weight of each score to allow for the best possible ranking performance, a grid search was performed. Each score was weighted from 0 to 100% in steps of 10%, leading to overall 3992 parameter configurations. The performance of each parameter configuration was measured using the MIBiG-based ranking benchmark.

### 4.7. Secondary Metabolite Prediction and Ranking Benchmark

Following the benchmark for SeMPI v1 [3], where the ranking of 40 BGC products was used as a metric to evaluate the pipeline performance, a novel benchmark was designed. For this benchmark, all PKS, NRPS, and PKS–NRPS hybrid BGCs in the MIBiG v2 with known products were collected. A total of 559 clusters were evaluated.

Two metrics were defined: First, the Tanimoto similarity (circular Morgan fingerprint, radius: 3) of the predicted scaffold with the known product of each cluster was computed. Second, all MIBiG v2 products of the benchmark BCGs were ranked based on the scoring metrics defined in Section 4.6.2. On the web server, the MCS-based and mixed-score-based rankings are only computed for preselected compounds, but for the benchmark, the scores were computed for all metabolites. In total, 828 metabolites were ranked, which is more than the total number of BGCs in the benchmark, since for some clusters, multiple possible products are described (e.g., abyssomicin C and atrop-abyssomicin C for BGC0000001). The rank of the true SM was used as a metric. Since the pipeline reports the Top 10 and Top 50 matching candidates, the percentage of true SMs ranked in this category was calculated.

To the best of our knowledge, the only other pipeline which produces mostly one predicted scaffold per BGC and is actively maintained is antiSMASH v5. We used the predicted scaffolds of antiSMASH v5 [16] as a comparison to our pipeline. For the natural compound ranking metric, the scoring scheme had to be adjusted, since antiSMASH v5 does not predict PSMs. Therefore, the comparative benchmark was performed on the predicted scaffolds without PSM ranking. The number of predicted BGCs for both pipelines was not the same for each cluster type. Multiple factors contributed to this discrepancy. First, the cluster type classification differs between pipelines. For example, SeMPI v2 classifies the clusters only based on the presence of functional modules, whereas we assume that the antiSMASH v5 classification is based on any biosynthetic domain found in the BGC. Second, in some rare cases, antiSMASH v5 would split the cluster query into multiple BGCs. These BGCs were excluded. Third, in some cases, either antiSMASH v5 or SeMPI v2 did not predict scaffolds. Nevertheless, for most BGCs, scaffolds were predicted by both pipelines. To show a fair comparison, a dataset was generated in which the predictions of both pipelines were merged. BGCs which were not predicted by either tool were excluded from this dataset. The cluster type classification from SeMPI v2 was used for all predictions. For some BGCs, antiSMASH v5 produced multiple cluster variants—for these instances, the mean of the similarity was taken.

Other tools are difficult to compare to our pipeline since the output characteristics are significantly different and hinder comparability. SBSPKS v2 [58] only predicts the products for domains encoded by one gene, not for entire clusters. Moreover, SBSPKS v2 can only process a maximum of 10 proteins via its web interface, which makes the processing of over 500 clusters very time-consuming. PRISM v3 produces a collection of scaffold permutations for most clusters. Our benchmark was designed to score single predicted scaffolds; hence, PRISM v3 was not used as a comparison. Moreover, we experienced various technical problems when submitting multiple BGCs in FASTA format to the PRISM v3 pipeline.

The benchmark was also used to determine the optimal weight of the three different scoring metrics. Therefore, a grid search was performed, where each of the scores was weighted differently.

### 4.8. Pipeline and Web Server Implementation

SeMPI v1 was designed as an extension to antiSMASH v3 [11], with the main goal of predicting PKs with deviations from the co-linearity principle as well as implementing a database screening in the StreptomeDB v2.0. To create an independent and flexible tool, SeMPI v2 was completely redesigned. SeMPI v2 performs the entire cluster prediction and scaffold generation independently.

#### 4.8.1. Input

The input must be provided in FASTA or GenBank format. The files can contain multiple records. SeMPI creates one result page for each record. If only DNA data are provided, the genes are predicted using Prodigal [59]. If the genes are already assigned, SeMPI can try to parse the genes and use them for further analysis.

#### 4.8.2. Cluster Prediction

The biosynthetic domains are detected using the profile-HMMs and thresholds described in Section 2.1. The proteins and their characteristics—such as position, gene locus, and strand direction—are transferred into a Pandas [60] DataFrame. The DataFrame allows very fast vectorized operations. The processing of an entire genome including database screening for similar natural compounds takes only a few minutes.

The modules are detected based on the rules described in Section 3.1. The most probable order of modules is generated using the module order algorithm described in Section 4.3. The substrates for each module are predicted using the RF models for AT- and A-domains. Based on the module characteristics and the substrate specificity, the corresponding building blocks are generated. These building blocks are combined into the putative metabolite scaffolds.

For each scaffold, a set of PSMs is generated using the profile-HMMs and regression models as described in Section 4.5.

#### 4.8.3. Metabolite Ranking

The predicted scaffolds are submitted to the database screening. The screening is performed using a user-selected choice of publicly available databases and/or a custom uploaded database (SDF or SMILES format). A custom database could be, for example, a set of bioactive samples that are assumed to be produced by a specific host. The screening can help to connect the samples with specific BGCs in the host.

The predicted scaffolds can also be modified and resubmitted to the natural compound screening. The molecular operations are performed using the JSME Molecule Editor [61]. This allows expert users to add certain modifications and substructures which are not yet correctly predicted. The database screening can also be used independently of the cluster prediction. Any molecule can be submitted, modified, and resubmitted via the web interface.

This feature also allows the usage of the SeMPI v2 database screening backend in combination with other BGC prediction tools such as PRISM v3 and antiSMASH v5.

#### 4.8.4. Web Server

The web server is built using Django [62] and a PostgreSQL database backend. Genomes are submitted via a simple input form. A real-time view shows the progress of the pipeline using Ajax [63,64]. The results are presented in the form of a data table [65]. The results include a summary page with detailed information about the domains and modules used to generate the clusters and an overview of the scaffolds for each BGC. Each BGC and also the entire genome can be investigated visually via the genome browser D3GB [66]. The browser shows the genes, domains, modules, blocks, and clusters.

## 5. Conclusions

The SeMPI v2 web server combines state-of-the-art predictions of PK and NRP scaffolds with a specialized screening in publicly available databases. This allows researchers to further connect the dots between BGCs and SMs. The information generated with SeMPI v2 is presented in a user-friendly web server frontend. The web server allows further customization of the predictions and thus more flexibility for expert users. BGCs can be observed in detail using the visually appealing integrated genome browser.

Since SeMPI v2 yields predictions that are complementary to existing pipelines, a comprehensive BGC analysis should include all available resources, such as antiSMASH v5, PRISM v3, SBSPKS v2, and others. Expert users can combine the generated structural features and submit multiple queries to the SeMPI v2 database screening backend to search for natural compound targets in different databases. In this role, SeMPI v2 contributes to further unraveling BGC mysteries and complements the link to putative SMs.

## Figures and Tables

**Table 1:** Cross-validation results and detection thresholds for biosynthetic domains used in the pipeline. The detection thresholds are referred to as domT by the HMMER manual [15].

Abbreviation	Name	F1	Precision	Recall	Support	Threshold
ACP	Acyl carrier protein	0.96	1.00	0.91	4315	13.9
AT	Acyltransferase	1.00	1.00	1.00	2893	47.4
A	Adenylation domain	1.00	1.00	0.99	3092	19.6
CAL	Coenzyme A ligase	0.94	0.89	1.00	180	221.8
C	Condensation domain	1.00	1.00	1.00	2734	25.9
DH2 ^1^	Dehydratase	0.98	0.95	1.00	414	33.2
DH	Dehydratase	0.99	1.00	0.99	1469	31.1
DHt ^2^	Dehydratase	0.88	0.98	0.81	113	44.3
ER	Enoylreductase	1.00	1.00	1.00	646	68.8
E	Epimerization domain	1.00	0.99	1.00	424	60.4
KR	Ketoreductase	1.00	1.00	1.00	3374	20
KS	Keto-synthase	1.00	1.00	1.00	3970	72
PCP	Peptide carrier protein	0.97	0.95	1.00	2785	21.9
TD	Reductive Thioesterase	1.00	1.00	1.00	118	42.1
TE	Thioesterase	1.00	1.00	1.00	824	36.5
bACP	β-branching acyl carrier protein	0.59	0.42	1.00	155	28.9
cMT	C-Methyltransferase	1.00	0.99	1.00	326	104.7
nMT	N-Methyltransferase	0.99	1.00	0.99	173	42.7
oMT	O-Methyltransferase	0.99	0.99	0.99	184	70.6
tAT_d	Trans-acyltransferase docking domain	0.98	0.97	1.00	665	50
Macro avg.		0.97	0.96	0.99	30,144	
Micro avg.		0.99	0.99	0.99	30,144	
Weighted avg.		0.99	0.99	0.99	30,144	

^1^ Specific dehydratase which is more commonly found in trans-AT PKS [16]. ^2^ Avoids false positive pyran synthase domain detection [16].

**Table 2:** The 20 most common modules found in the MIBiG. The functional classification, normal (N), special (S), and not functional (NF) is explained in Section 4.2. The modification refers to the reduction profile of PKS modules or epimerization of NRPS modules. The occurrence refers to the overall count in all BGCs of the MIBiG v2.

Domain Order	Functional	Type	Modification	Occurrence
C-A-PCP	N	NRPS	L	1429
KS-AT-DH-KR-ACP	N	PKS	Enoyl	895
KS-AT-KR-ACP	N	PKS	Hydroxyl	628
C-A-PCP-E	N	NRPS	E	362
KS-AT-DH-ER-KR-ACP	N	PKS	Alkyl	335
KS-AT-ACP	N	PKS	Ketone	247
A-PCP	N	NRPS	L	235
C-A-PCP-TE	N	NRPS	L	202
KS-tAT_d-KR-ACP	S	PKS	Hydroxyl	139
KS-tAT_d-DH-KR-ACP	S	PKS	Enoyl	125
C-A-nMT-PCP	N	NRPS	L	120
KS-tAT_d-ACP	S	PKS	Alkyl	71
cAL-ACP	NF	PKS	-	62
KS-AT-DH-KR-ACP-TE	N	PKS	Enoyl	61
C-A-ACP	N	Mixed	L	61
C-PCP	NF	NRPS	-	59
KS-ACP	S	PKS	Ketone	52
KS-tAT_d	S	PKS	Ketone	52
KS-AT	S	PKS	Ketone	49
KS-tAT_d-DH-KR-cMT-ACP	S	PKS	Enoyl	49

**Table 3:** Best grid search parameters for the RF models for AT- and A-domain specificity classification. The error for the micro F1-score is based on the standard deviation over all cross-validation queries.

Parameter	A-Domains	AT-Domains
Max. depth	100	50
Max. features	auto	auto
N. estimators	1000	100
Min. samples split	10	1
Bootstrap	No	No
Criterion	Gini	Gini
Min. samples leaf	1	2
F1-score	0.75 ± 0.05	0.96 ± 0.02

**Table 4:** Performance for each substrate for AT-domain classification.

Substrate	F1-Score	Precision	Recall	Support
Ethylmalonyl	0.5	1	0.33	15
Malonyl	0.98	0.99	0.98	278
Methoxymalonyl	0.59	1	0.42	12
Methylmalonyl	0.94	0.9	0.99	194
Micro avg.	0.95	0.95	0.95	499
Macro avg.	0.75	0.97	0.68	499

**Table 5:** Performance for each substrate for A-domain classification. The amino acids are named according to the standard amino acid one-letter code. Special substrate abbreviations are: 2-amino-adipic-acid (aad), beta-hydroxy-tyrosine (bht), diaminobutyric acid (dab), 2,3-dihydroxy-benzoic acid (dhb), 2,3-dehydroaminobutyric acid (dhbu), 3,5-dihydroxy-phenyl-glycin (dhpg), Hydroxy-L-ornithine (horn), 4-hydoxy-phenyl-glycine (hpg) ornithine (orn), pipecolic acid (pip).

Substrate	F1-Score	Precision	Recall	Support
A	0.91	0.92	0.90	395
C	0.83	0.79	0.88	64
D	0.75	0.75	0.75	56
E	0.46	0.7	0.35	55
F	0.40	0.32	0.54	122
G	0.77	0.73	0.82	78
H	0.15	0.33	0.10	10
I	0.77	0.86	0.70	63
K	0.36	0.62	0.25	20
L	0.70	0.73	0.67	149
N	0.75	0.74	0.76	55
P	0.76	0.79	0.74	61
Q	0.84	0.84	0.84	37
R	0.56	0.63	0.50	24
S	0.77	0.76	0.79	132
T	0.83	0.78	0.89	118
V	0.72	0.68	0.76	119
W	0.77	0.79	0.76	41
Y	0.50	0.64	0.42	72
aad	0.82	0.88	0.78	63
bht	0.48	0.43	0.55	11
dab	0.88	0.92	0.84	44
dhb	0.97	0.96	0.99	155
dhbu	0.27	0.50	0.18	11
dhpg	1.00	1.00	1.00	12
horn	0.73	0.80	0.67	12
hpg	0.91	0.89	0.93	42
orn	0.58	0.67	0.52	31
pip	0.60	0.86	0.46	13
Micro avg.	0.76	0.76	0.76	2065
Macro avg.	0.69	0.74	0.67	2065

**Table 6:** Performance metrics for the classification of AT- and A-domain substrates. ROC-AUC refers to the area under the receiver operating characteristic curve.

Metric	AT-Domains	A-Domains
Accuracy	0.95	0.76
Error rate	0.05	0.24
Matthews correlation coefficient (MCC)	0.91	0.74
ROC-AUC (macro)	1.00	0.96
ROC-AUC (micro)	1.00	0.97
Precision (macro)	0.97	0.74
Precision (micro)	0.95	0.76
Recall (macro)	0.68	0.67
Recall (micro)	0.95	0.76
F1-score (macro)	0.75	0.69
F1-score (micro)	0.95	0.76
Support	499	2065

**Table 7:** Selected Pfam domains and corresponding PSMs. The number of appearances of a certain PSM in the structure of a secondary metabolite is correlated to the number of Pfam domains found in the producing cluster. The descriptions are derived from the Pfam database. Pfam-derived abbreviations are: deoxythymidine diphosphate (dTDP), nicotinamide adenine dinucleotide (NAD), nucleoside diphosphate (NDP), uridine diphosphate glucose (UDP), guanosine diphosphate (GDP), flavin adenine dinucleotide (FAD), domain of unknown function (DUF).

PSM	Pfam ID	Description	Pearson Correlation
Glyco	PF00908.16	dTDP-4-dehydrorhamnose 3,5-epimerase	0.67
Glyco	PF01370.20	NAD dependent epimerase/dehydratase family	0.64
Glyco	PF03559.13	NDP-hexose 2,3-dehydratase	0.61
Glyco	PF00201.17	UDP-glucoronosyl and UDP-glucosyl transferase	0.59
Glyco	PF03033.19	Glycosyltransferase family 28 N-terminal domain	0.56
Glyco	PF01041.16	DegT/DnrJ/EryC1/StrS aminotransferase family	0.52
Glyco	PF08421.10	Putative zinc binding domain	0.47
Glyco	PF16363.4	GDP-mannose 4,6 dehydratase	0.44
Glyco	PF04101.15	Glycosyltransferase family 28 C-terminal domain	0.28
Glyco	PF01075.16	Glycosyltransferase family 9 (heptosyltransferase)	0.21
Glyco	PF00728.21	Glycosyl hydrolase family 20, catalytic domain	0.18
Glyco	PF02838.14	Glycosyl hydrolase family 20, domain 2	0.18
Glyco	PF01915.21	Glycosyl hydrolase family 3 C-terminal domain	0.13
Glyco	PF00933.20	Glycosyl hydrolase family 3 N terminal domain	0.13
Glyco	PF14885.5	Hypothetical glycosyl hydrolase family 15	0.1
Cl	PF04820.13	Tryptophan halogenase	0.66
Cl	PF00999.20	Sodium/hydrogen exchanger family	0.51
Spiroketal	PF12680.6	SnoaL-like domain	0.61
Spiroketal	PF00890.23	FAD binding domain	0.54
SS	PF07992.13	Pyridine nucleotide-disulphide oxidoreductase	0.46
NO_2_	PF01678.18	Diaminopimelate epimerase	0.67
NO_2_	PF06722.11	Protein of unknown function (DUF1205)	0.27
6-Ring	PF16197.4	Ketoacyl-synthetase C-terminal extension	0.53
6-Ring	PF02801.21	Beta-ketoacyl synthase, C-terminal domain	0.52
6-Ring	PF08990.10	Erythronolide synthase docking	0.51
6-Ring	PF00743.18	Flavin-binding monooxygenase-like	0.46
5-Ring	PF12680.6	SnoaL-like domain	0.56
5-Ring	PF00890.23	FAD binding domain	0.53
5-Ring	PF01551.21	Peptidase family M23	0.46
5-Ring	PF08990.10	Erythronolide synthase docking	0.45
5-Ring	PF00486.27	Transcriptional regulatory protein, C terminal	0.26
5-Ring	PF16197.4	Ketoacyl-synthetase C-terminal extension	0.26
5-Ring	PF00109.25	Beta-ketoacyl synthase, N-terminal domain	0.26

**Table 8:** R^2^-score (coefficient of determination) for the prediction of PSMs based on 5-fold cross-validation. The baseline is based on a hypothetical model, assuming that no PSM is predicted at all.

PSM Name	Baseline Model	Regression Model
Glyco	−0.118	0.548
Cl	−0.091	0.26
Spiroketal	−0.031	0.427
SS	−0.02	0.126
NO_2_	−0.015	0.097
6-Ring	−0.14	0.416
5-Ring	−0.052	0.374

**Table 9:** Average (arithmetic mean) Tanimoto similarity between produced SMs and the predictions made by SeMPI v2 and antiSMASH v5.

Cluster Type	Number of Clusters	SeMPI v2	AntiSMASH v5
PKS	110	0.44	0.42
NRPS	158	0.57	0.50
Mixed	219	0.45	0.42
All	487	0.48	0.45

**Table 10:** Best scoring parameter weights for each BGC type. The corresponding percentage of Top 10 and Top 50 ranked instances of the true SMs are shown.

Type	Similarity-Score	MCS-Score	PSM-Score	Top 10	Top 50
PKS	1	1	0.3	0.53	0.72
NRPS	1	0.4	0.5	0.59	0.72
Mixed	0.4	0.6	0.1	0.45	0.64
All	0.3	0.4	0.1	0.47	0.67

**Table 11:** Percentage of Top 10 and Top 50 ranked instances of true SMs based on different scoring functions. The mixed scoring is based on the parameter weights determined in Section 2.5.2.

Scoring Function	Cluster Type	SeMPI v2 (Top 10)	AntiSMASH v5 (Top 10)	SeMPI v2 (Top 50)	AntiSMASH v5 (Top 50)
Similarity	PKS	25.4	20.3	45.61	48.55
MCS	PKS	36.0	22.5	56.14	49.28
Mixed	PKS	49.1	-	72.81	-
Similarity	NRPS	50.3	45.9	64.57	61.01
MCS	NRPS	45.1	45.9	57.71	61.01
Mixed	NRPS	55.4	-	68.57	-
Similarity	Mixed	25.1	22.6	46.91	41.03
MCS	Mixed	32.5	22.6	47.74	41.03
Mixed	Mixed	39.9	-	62.14	-
Similarity	All	33.5	29.5	52.44	49.59
MCS	All	37.4	30.1	52.82	49.80
Mixed	All	47.0	-	66.54	-

**Table 12:** RF parameters and their configuration used for the grid search to determine the best configuration for substrate specificity prediction. Each parameter is described in detail in the scikit-learn documentation [48].

Parameter	Configurations
Max. depth	10, 50, 100, None
Max. features	1, 3, 10, auto, sqrt
N. estimators	100, 500, 1000
Min. samples split	2, 5, 10
Bootstrap	True, False
Criterion	gini, entropy
Min. samples leaf	1, 5, 10

**Table 13:** SMARTS patterns used to match PSMs in known BGC products described in the MIBiG.

Name	SMARTS Patterns
Glyco	[#6]O[D3]1CCCCO1
Cl	Cl
Spiroketal	[C&R]O[C&R]([C&R])([C&R])O[C&R]
SS	SS
NO_2_	O=[N+][O−]
6-Ring	CC1CCCC(C)O1
5-Ring	C1CCCO1

**Table 14:** Databases and the number of stored molecules used for the screening of predicted scaffolds. All compounds are linked to their source database. The predicted streptomyces SMs are linked to the preprocessed database on the SeMPI v2 web server.

Database Name	Number of Molecules
MIBiG	1528
StreptomeDB 2.0	3990
NANPDB	6841
DrugBank	9277
MolPort NP	120,555
Analyticon Zinc	663
ChEBI (3star)	46,547
Norine	641
Predicted streptomyces SMs	1519

## Data Availability

The data presented in this study is available in the supplementary material. The preprocessed databases are available on the web server.

## Supplementary Materials

The following are available online at https://www.mdpi.com/2218-1989/11/1/13/s1, Table S1: Complete module analysis of the MIBiG (Module_Analysis.csv), Table S2: Adenylation domain sequences used to train the random forest models (A_domains.csv), Figure S1: PKS reduction pattern demonstrated on the abyssomicin BGC (abyssomicin_prediction_comparison.docx).
