# Supplementary material for: SeMPI 2.0—A Web Server for PKS and NRPS Predictions Combined with Metabolite Screening in Natural Product Databases
Source: Metabolites. 2020 Dec 29;11(1):13. doi: 10.3390/metabo11010013 (PMC7823522; doi:10.3390/metabo11010013)
Supplement: Supplementary file 1 [file metabolites-11-00013-s001.zip › abyssomicin_prediction_comparison.docx]

**Supplementary Materials: SeMPI 2.0 - A web server for PKS and NRPS predictions combined with metabolite screening in natural product databases**


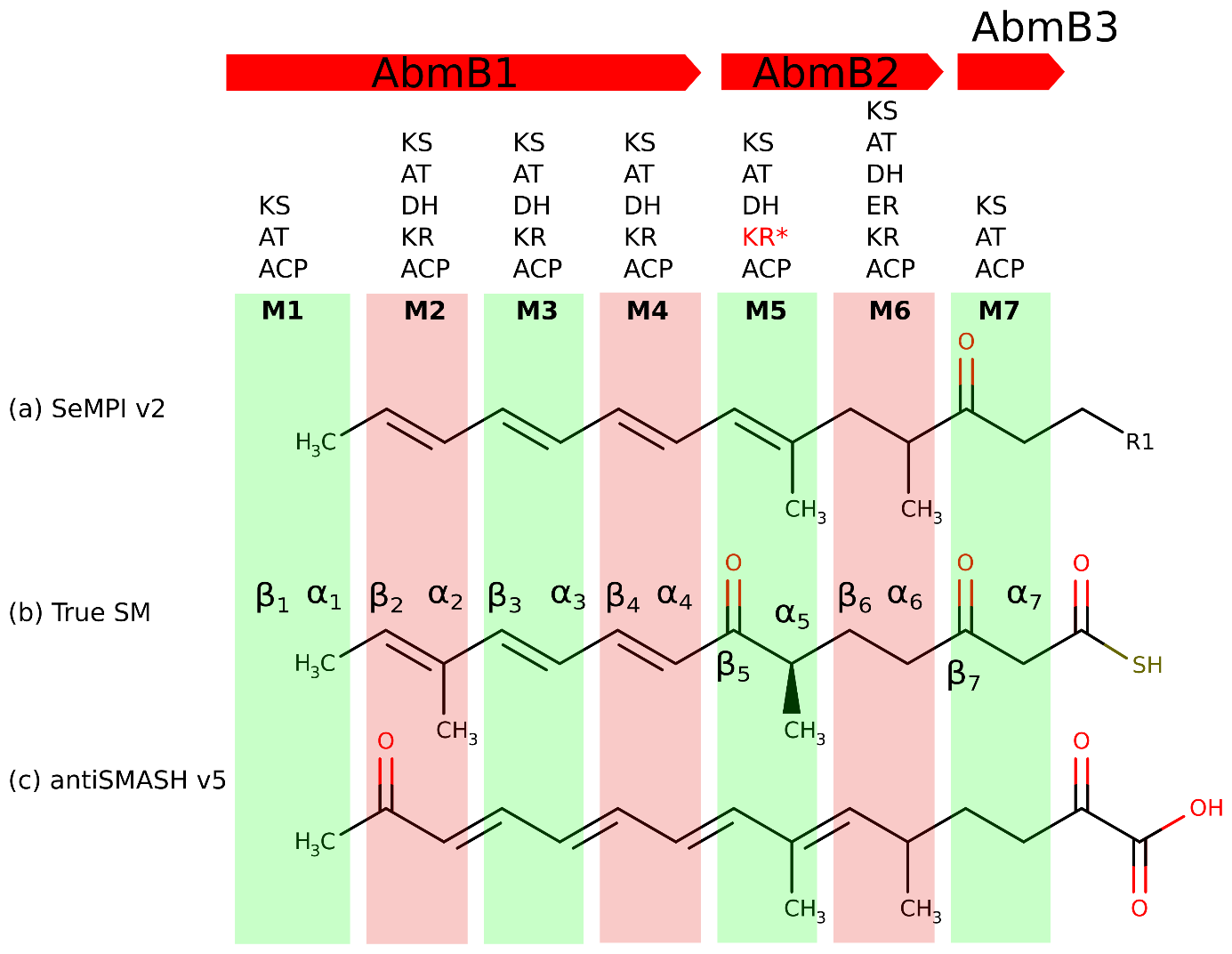


**Figure 1.** Comparison of SM predictions for the abyssomicin BGC made by SeMPI v2 and antiSMASH v5 with the produced SM. The top panel shows the encoding genes and the domain composition of each module. The KR domain of the 5th module is inactivated. The SeMPI v2 prediction is shown on top (a). The produced core secondary metabolite is derived from the article by Tu et al. [1] and drawn in the middle (b). The indices of the α- and β-position correspond to the module which performs the reduction. The antiSMASH v5 prediction was taken from the MIBiG database (accessed: 22.07.2020) and is shown at the bottom (c). antiSMASH v5 assigned the reduction pattern with an offset. Also, in the cases where the reduction to a double bond was performed, the double bond was assigned at the wrong position.

1. Tu, J.; Li, S.; Chen, J.; Song, Y.; Fu, S.; Ju, J.; Li, Q. Characterization and heterologous expression of the neoabyssomicin/abyssomicin biosynthetic gene cluster from *Streptomyces koyangensis* SCSIO 5802. *Microb. Cell Fact.* **2018**, *17*, 28, doi:10.1186/s12934-018-0875-1.
